# Antioxidant and Antiproliferative Activities of Phenolic Extracts of *Eriobotrya japonica* (Thunb.) Lindl. Fruits and Leaves

**DOI:** 10.3390/plants12183221

**Published:** 2023-09-10

**Authors:** Agata Maria Pawłowska, Natalia Żurek, Ireneusz Kapusta, Marinella De Leo, Alessandra Braca

**Affiliations:** 1 Department of Food Technology and Human Nutrition, College of Natural Sciences, University of Rzeszow, 4 Zelwerowicza St., 35-601 Rzeszow, Poland; agpawlowska@ur.edu.pl (A.M.P.); nzurek@ur.edu.pl (N.Ż.); ikapusta@ur.edu.pl (I.K.); 2 Department of Pharmacy, University of Pisa, Via Bonanno Pisano 33, 56126 Pisa, Italy; alessandra.braca@unipi.it

**Keywords:** loquat, *Eriobotrya japonica*, *Rhaphiolepis bibas*, fruits, leaves, antioxidant activity, antiproliferative activity, polyphenols

## Abstract

Increasing interest in new sources of secondary metabolites as biologically active substances has resulted in an advanced study of many plant species. Loquat (*Eriobotrya japonica* (Thunb.) Lindl. = *Rhaphiolepis bibas* (Lour.) Galasso & Banfi, Rosaceae family), an evergreen, subtropical fruit tree, native to China and Japan, but cultivated in southern countries of Europe, is a species commonly used in folk medicine and may be an excellent source of bioactive compounds. Therefore, the aim of the present study was to evaluate the profile of the phenolic constituents of *E. japonica* fruits and leaves originating from Tuscany (Italy), as well as their in vitro antioxidant and chemopreventive activities on human cancer cell lines breast adenocarcinoma (MCF-7), colon adenocarcinoma (Caco-2 and HT-29), and glioblastoma (U87MG). Results revealed that the extract of leaves displayed higher antioxidant and anticancer potential than the fruit extract and contained 25 individual phenolic compounds that have been characterized and quantified by the UPLC-PDA-MS method. The antiproliferative activity was correlated with the content of polyphenolic compounds indicating that both fruits and leaves are a good source of antioxidants and may be exploited as nutraceuticals enriching food or as components for the cosmetic/pharmaceutical industry.

## 1. Introduction

The loquat (*Eriobotrya japonica* (Thunb.) Lindl. = *Rhaphiolepis bibas* (Lour.) Galasso & Banfi) is an evergreen, subtropical fruit tree [1], native to China and Japan, belonging to the Rosaceae family. Its cultivation, however, has spread almost all over the world and it can be found in countries such as Spain, India, Egypt, Cyprus, Italy, Australia, Mexico, and Tunisia [2]. The plant has about 800 varieties, divided according to the countries where they are grown [3]. *E. japonica* may occur as a shrub, which usually reaches 3 to 4 m in height, or as a tree, which can reach up to 10 m. It is characterized by an extremely short trunk with a rounded crown. Leaves are arranged alternately on the stem and are covered with hairs on the upperside and have a yellow-brown color on the underside. Their length is estimated at approx. 10–25 cm [4]. The fruits are of yellow and orange colors, spherical in shape, and their diameter is 3–5 cm. The flesh is distinguished by juiciness and a slightly sour aftertaste [5]. Loquat contains only 47 kcal/100 g of fruits, but depending on the variety, *E. japonica* is a rich source of organic acids, vitamins, and minerals [6,7]. In addition, it is an outstanding source of various bioactive compounds, including phenolic acids, flavonoids, carotenoids, and triterpenoids, which have various biological roles, i.e., antioxidant, antiviral, anticancer, hypoglycemic, anti-inflammatory, cytotoxic, antimutagenic, and hypolipidemic effects [8,9].

On the other hand, *E. japonica* performs the function of a medicinal plant. Its leaves have been used in Traditional Chinese Medicine since ancient times [10] to treat respiratory system diseases [11], chronic irritation of the digestive system, skin inflammations [12], and diabetes [13]. Moreover, studies on various extracts of loquat have shown that they have strong antioxidant abilities [14]. Out of the 56 selected Chinese plants, the loquat leaf showed greater antioxidant capacity than 54 other medicinal plants [15]. Similarly, some lines of data proved that *E. japonica* leaves also exhibit cytotoxic activity [16,17]. Loquat tea inhibited the proliferation of human promyelocytic leukemia cells [16] and a water-soluble portion of the extract was active against two human oral tumor cell lines [17]. However, the phytonutrient composition of extracts from different parts of this plant varies distinctly [18]. Significant discrepancies in the chemical composition of the plant are also related to their geographical origin [19,20].

Therefore, the aim of this study was to evaluate the secondary metabolite profile and biological properties of phenolic extracts of fruits and leaves of *E. japonica* originating from Italy, in order to draw attention to this species and to contribute to the improvement in the potential value of this minor fruit as food as well as the exploitation of leaves as a new, alternative source of antioxidants and nutraceuticals, and their application possibilities in the food-pharma industry. For this purpose, the antioxidant activity was investigated by using five different methods, and the chemopreventive potential was evaluated for the first time on four human cancer cell lines, such as breast adenocarcinoma (MCF-7), colon adenocarcinoma (Caco-2, and HT-29), and glioblastoma (U87MG).

## 2. Results and Discussion

### 2.1. Total Phenolic, Flavonoid, and Proanthocyanidin Contents

The total contents of phenolic compounds (TPC), flavonoids (TFC), and proanthocyanidins (TPA) in the fruits and leaves of *E. japonica* are presented in Table 1.

The leaves of the plant showed higher content of the three analyzed groups of compounds than the fruits. The TPC in the obtained leaf extract was 6.05 mg gallic acid equivalent (GAE)/100 g dry weight (dw), TFC 1.23 mg quercetin equivalent (QE)/100 g dw, and TPA 1.19 mg cyanidin chloride (CYE)/100 g dw.

The TPC and TFC in loquat fruit extracts, according to Kaur et al. [20], were 8.16 mg/GAE 100 g and 20.92 mg rutin equivalent (RE)/100 g, respectively, and according to Xu and Chen, they ranged from 24.05 to 57.23 mg GAE/100 g and from 2.12 to 7.75 mg RE/100 g for 12 plant varieties [21]. In contrast, the phenolic content of some cultivars grown in Italy was found to be below 1 mg GAE per 100 g of sample [19].

In the leaf extract, the TPC in the work of Mogole et al. was estimated to be at the level of 123.3 to 381.0 mg GAE/100 g, and Bisso et al. reported the quantities of polyphenols and flavonoids to be 98.00 mg GAE/100 g and 38.03 mg QE/100 g, respectively [22,23].

TPA in loquat fruits and leaves has not been analyzed in other studies so far. Results showed that leaves contain a five times higher content of proanthocyanidins than fruits.

### 2.2. Antioxidant Activity

The antioxidant activity assays of *E. japonica* fruit and leaf extracts were assessed using five methods, such as ABTS^•+^ radical scavenging activity, reduction of copper ions (CUPRAC), ferrous ions chelating capacity (ChA), scavenging activity of superoxide (O_2_˙^−^), and hydroxyl radicals (OH˙). The results are listed in Table 2.

Using all the performed tests, a higher antioxidant activity was revealed for the leaves of the plant. For the ABTS test, the obtained value was 74.35 mmol Trolox equivalent (TE)/100 g dw; for the CUPRAC test, it was 62.01 mmol TE/100 g dw; and for the ChA, O_2_˙^−^, and OH^−^ tests—941.25, 244.30, and 661.04 µg/mL, respectively. In turn, the antioxidant activity of *E. japonica* fruits, expressed by the ABTS and CUPRAC methods, equaled 17.38 and 14.57 mmol TE/100 g dw. For the ferrous ion chelating capacity, scavenging activity of superoxide, and hydroxyl assays, the obtained values were outside the range of the assay.

Xu and Chen observed lower ABTS scavenging activity for twelve varieties of *E. japonica* fruit [21]. The values noted by them ranged from 0.13 to 0.33 mmol TE/100 g. On the other hand, Mokdad-Bzeouich et al. showed a higher iron ion chelating capacity than in our study (540 µg/mL) but a lower capture of superoxide radicals (45 µg/mL) and hydroxyl radicals (620 µg/mL) [24].

The statistical analyses also revealed strong correlations between antioxidant activity and selected groups of polyphenolic compounds (TPC vs. ABTS, r > −0.957, *p* < 0.01; TPC vs. ChA, r > 0.935, *p* < 0.01 in the leaf extract and TPC vs. ABTS, r > −0.992, *p* < 0.01; TPF vs. CUPRAC, r > 0.918, *p* < 0.01, TPA vs. CUPRAC, r > −0.998, *p* < 0.05) (see Table 3 and Table 4). This demonstrates that both extracts can be exploited as an easily available source of polyphenolic compounds, as well as a source of natural antioxidants.

### 2.3. Cell Viability

**Figure 1:**
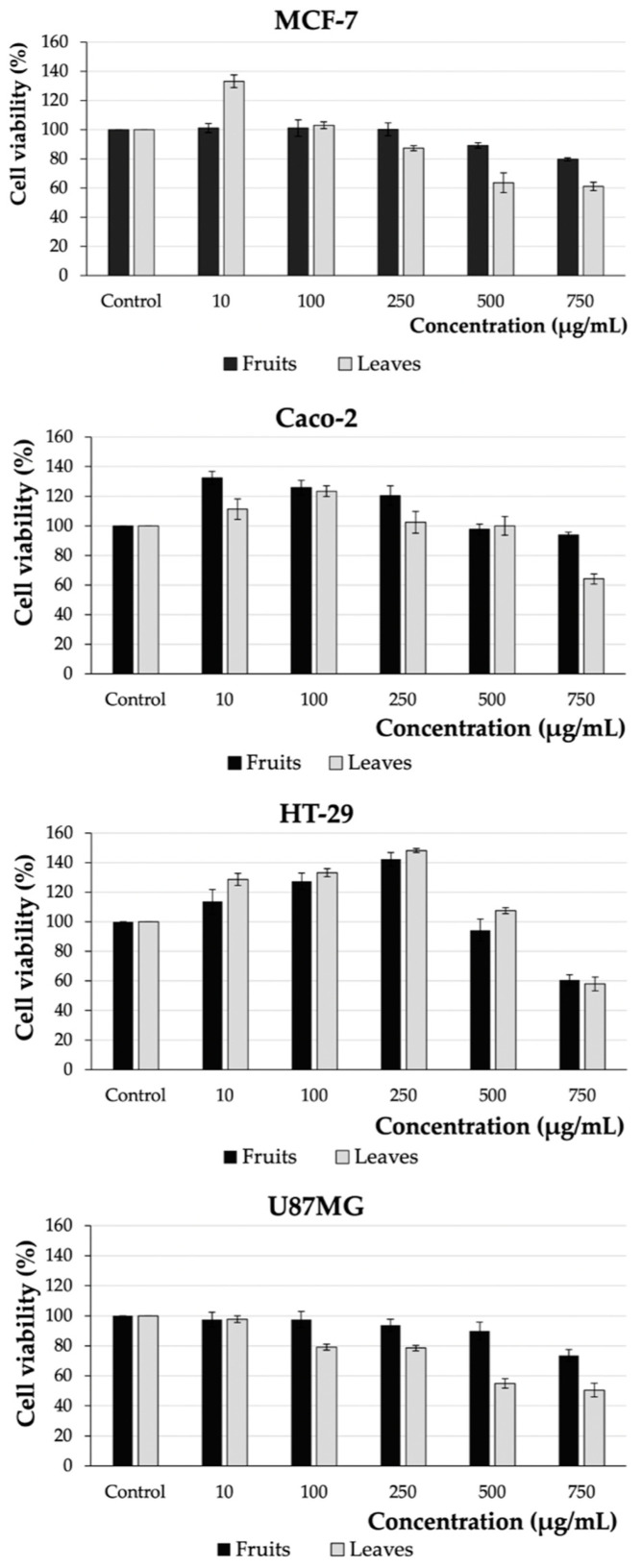
Effect of fruit and leaf *Eriobotrya japonica* extracts on the viability of breast adenocarcinoma (MCF-7), Caco-2, HT-29 (colon adenocarcinoma), and U87MG (glioblastoma) cell lines. The cells were treated for 48 h with the extracts at five increasing concentrations (10–750 µg/mL). Graphs represent mean values ± SD from three independent experiments.

The cytotoxic effects of fruit and leaf *E. japonica* extracts were tested on four cancer lines (MCF-7, Caco-2, HT-29, and U87MG) by means of the MTS test. Cells were treated for 48 h with increasing doses of the extracts (10–750 µg/mL). Untreated cells as well as cells treated with ethanol at concentrations of 0.006–0.45% were used as controls (see Figure S1). The results of the tests are presented in Figure 1.

The analyzed extracts impacted the viability of cells depending on the concentrations used. The highest concentration of *E. japonica* leaf extract (750 µg/mL) showed the greatest cytotoxic effect on glioblastoma cells (U87MG) and the lowest on colorectal adenocarcinoma cells (Caco-2). The viability of both lines was reduced by 49.50% and 35.88%, respectively. In contrast, loquat fruit extract showed a less pronounced effect on cell viability. The highest cytotoxic effect at a concentration of 750 µg/mL was shown for colorectal adenocarcinoma cells, HT-29 (39.37%), and the lowest for MCF-7 breast adenocarcinoma cells (20.24%).

So far, few reports have been published on the anticancer activity of *E. japonica* fruits and leaves. Significantly higher anticancer activity was reported for loquat fruits by Abdel Raoof et al. [25]. They obtained IC_50_ values equal to 0.012 µg/mL for ovarian cancer (SKOV-3), 35.00 µg/mL for prostate adenocarcinoma (PC-3), and 1.53 µg/mL for hepatocellular carcinoma (HEPG-2). On the other hand, Alwash, in the evaluation of *E. japonica* fruit juice against two cell lines of cervical cancer (Hela) and rhabdomyosarcoma (RD), showed only a slight decrease in their viability by 10.09% and 17.78%, respectively (at a concentration of 500 µg/mL) [26].

Instead, for the leaves of *E. japonica*, the anticancer effect was evaluated against a promyelocytic leukemia cell line (HL-60). The loquat tea at a concentration of 100 µg/mL, decreased the viability of the cells to 58% by inducing cell apoptosis, which was characterized by DNA fragmentation, activation of caspase-3, -8, and -9, and inactivation of poly(ADP)ribose polymerase (PARP1) [16]. In turn, another study showed that mainly oligomeric proanthocyanidins isolated from *E. japonica* leaves are responsible for the antiproliferative activity of the studied extract [17].

The antiproliferative activity was correlated with the content of polyphenols. Statistical analysis showed strong correlations between the cytotoxic activity of the cell lines and both, groups of polyphenolics (HT-29 vs. TFC, r > 0.999, *p* < 0.05 in the leaf extract and HT-29 vs. TFC, r > 0.974, *p* < 0.05), as well as individual phenolic compounds (HT-29 vs. Flavones, r > −0.995, *p* < 0.05 in the leaf extract and Caco-2 vs. Phenolic acids, r > 0.999, *p* < 0.05; MCF-7 vs. Flavones, r > −0.999, *p* < 0.05) (see Table 3 and Table 4).

### 2.4. Phenolic Compound of Fruit and Leaf Extracts

**Figure 2:**
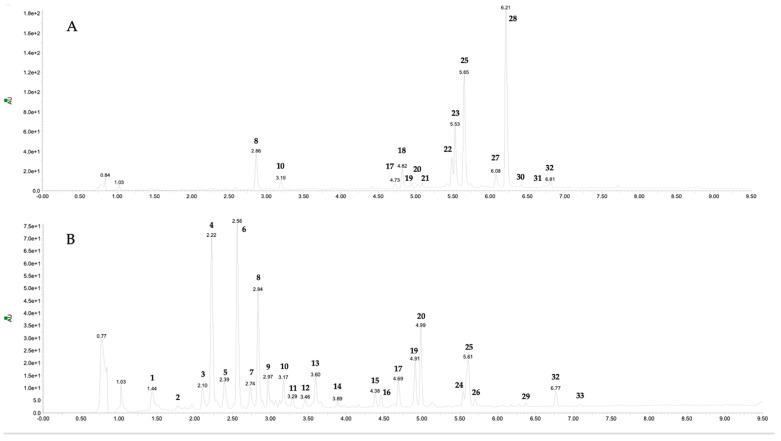
Photodiode Array (PDA) chromatogram of *Eriobotrya japonica* fruit (**A**) and leaf (**B**) extracts recorded at the wavelength of 350 nm. Peak data are shown in Table 5.

The determination of individual phenolic compounds of alcoholic extracts of fruits and leaves of *E. japonica* was performed using the Ultra-Performance Liquid Chromatography Photodiode Array Detector and Mass Spectrometry (UPLC-PDA-MS/MS) method. The tentative identification of the compounds was carried out by comparison of their retention times, elution orders, UV–Vis, and MS spectra with available literature. The target identification was achieved by the use of authentic standards. All the spectroscopic and chromatographic data are listed in Table 5. The LC-MS chromatograms of the fruit and leaf extracts are presented in Figure 2.

The extract prepared from the fruits contained 15 phenolic compounds in its composition; in turn, the leaf extract consisted of 25 constituents. In total, in both extracts, 33 different components were found.

Components **10**, **14**, **18**, **24**, and **25** were recognized by comparison of their chromatographic and spectrometric data with commercially available samples as caffeic acid (**10**), quercetin 3-*O*-xyloside (**14**), kaempferol 3-*O*-rutinoside (**24**), and rosmarinic acid (**25**). Compounds **4**, **7–9**, **13**, **16**, **23**, **29**, and **33** were previously detected in this plant and, thus, identified by comparison of their UV–Vis and MS spectra with available literature as neochlorogenic acid (**4**), 3-*O*-coumaroylquinic acid (**7**), chlorogenic acid (**8**), cryptochlorogenic acid (**9**), ferulic acid (**13**), luteolin 7-*O*-malonylglucoside (**23**), dicaffeoyl quinic acid (**29**), and feruloylquinic acid (**33**) [1,17,18,27,28,29]. Compounds **1–3**, **5**, **6**, **11**, **12**, **17**, **26**, **31**, and **32** were predicted to be caffeic acid esters. They all exhibited typical UV spectra with two maximum peak absorption bands at 200 sh and 325–330 nm and a distinctive fragmentation pathway with a fragment ion at *m/z* 179 [30]. Component **19** showed the parent ion and daughter ion at *m/z* 549 and *m/z* 505 and 301, respectively. It indicated the presence of malonylglucoside in conjunction with quercetin aglycone, and thus this compound was identified as quercetin 3-*O*-malonylglucoside. The presence of quercetin derivatives in loquat fruits has been widely reported [4,25,29]. Compound **20** was distinguished as chrysoeriol rutinoside. It showed a precursor ion of *m/z* 607 and the fragment ion of *m/z* 299 indicating the loss of a rhamnose and a glucose moiety from the aglycon tail. The aglycon performed the same spectrometric data as component **30**, and thus it was recognized as chrysoeriol. Chrysoeriol glycosides have been previously revealed in different parts of loquat [29]. Compound **21** was potentially identified as salvianolic acid B, based on the [M − H]^−^ ion at *m/z* 717, and the daughter ion at *m/z* 339 in the MS/MS spectrum, as reported by Cheng et al. [31]. Components **22**, **27**, and **28** all gave the deprotonated aglycone fragment at *m/z* 269, suggesting that they originated from apigenin. Compound **22** was recognized as apigenin hexoside. It showed the parent ion at *m/z* 431 and the loss of 162 u. It is difficult, however, to identify the isomers of sugar moieties by mass spectrometry alone [32]. Constituents **27** and **28** yielded the [M − H]^−^ 42 u higher than compounds **22**, suggesting the occurrence of an acetyl part in the molecule and, thus, being identified as apigenin acetylhexosides. Indeed, the presence of derivatives of apigenin in the fruits and leaves of *E. japonica* has already been defined [4,29]. Components **15** remains unidentified; however, its relatively low molecular weight, and a UV spectrum with maximal absorbance at a wavelength of 295 nm, led us to classify it in the phenolic acids group.

The total amount of polyphenols (Table 5) in *E. japonica* fruits was 9.285 mg/100 g dw. Apigenin acetylhexoside was the predominant compound (31.52%), followed by rosmarinic acid (21.87%). Interestingly, despite the composition being widely investigated, these compounds were not reported in the available literature as loquat constituents. It is well-known that the chemical composition of the plant may vary depending on species, season, growing conditions, and genetic differences [33]. Instead, the content of polyphenols in the leaf extract was at the level of 26.796 mg/100 g dw. Caffeic acids occurred in the leaves most abundantly, which is in accordance with previously published data [4,19].

## 3. Materials and Methods

### 3.1. Materials and Reagents

Quercetin (≥95% purity), gallic acid (≥98% purity), cyanidin chloride (≥98% purity), β-nicotinamide adenine dinucleotide, ≥97% purity (NADH), thiobarbituric acid, trichloroacetic acid, ascorbic acid, nitro blue tetrazolium (NBT), phenazine methosulfate (PMS), hydrogen peroxide, dulbecco’s modified eagle medium (DMEM), antibiotics (penicillin, and streptomycins), fetal bovine serum, 0.25% trypsin-EDTA, phosphate-buffered saline were purchased from Sigma-Aldrich (Steinheim, Germany). MTS assay test was purchased from Promega (Madison, WI, USA). Reference standard compounds for UPLC analyses were obtained from Extrasynthese (Lyon, France) and Sigma-Aldrich (Darmstadt, Germany). All other chemicals were from Chempur (Piekary Śląskie, Poland).

### 3.2. Plant Material and Extract Preparation

*Eriobotrya japonica* leaves and fruits were collected in Pisa, Italy, in May 2021, and identified by one of the authors (M. De Leo). A voucher specimen (N° PI063762) was deposited at the Herbarium Horti Botanici Pisani (Pisa, Italy).

Fresh fruits (292.7 g) and leaves (398.2 g) were lyophilized, grounded, and extracted with 70% ethanol using an ultrasonic bath for 30 min at 30 °C, centrifuged at 10,000× *g* for 10 min, and the obtained supernatants were used for further analysis. For evaluation of cell viability, extracts were obtained using 30% ethanol following the procedure described above.

### 3.3. Determination of Total Phenolic, Flavonoid, and Total Proanthocyanidin Content

The total phenolic content (TPC) was evaluated using the method described by Gao et al. [34]. An amount of 2 mL of water, 0.2 mL of Folin–Ciocalteu solution, and 1.0 mL 20% sodium carbonate were added to the plant extracts. After 1 h, the absorbance was measured at 765 nm using a UV–VIS spectrometer (Type UV2900, Hitachi, Japan).

The total flavonoid content (TFC) was evaluated using the method described by Chang et al. [35]. The plant extracts were mixed with 0.2 mL 10% aluminum chloride, 3.0 mL ethanol, 0.2 mL 1 M sodium acetate, and 5.2 mL water. After 30 min, the absorbance was measured at 415 nm.

The total proanthocyanidin content (TPA) was determined using the method described by Żurek et al. [36]. The plant extracts were mixed with 3 mL *n*-BuOH with 35% HCl (95:5) and 0.1 mL of 2% iron (III) ammonium sulfate in 2 M HCl. The samples were incubated at 95 °C for 45 min, then cooled and the absorbance was measured at 550 nm.

The results of TPC, TFC, and TPA contents were expressed in mg equivalent of gallic acid per g of dw (mg GAE/100 g dw), mg equivalent of quercetin (mg QE/100 g dw), and mg equivalent of cyanidin chloride (mg CYE/100 g dw), respectively.

### 3.4. Determination of Polyphenols Profile by UPLC-Q-TOF-MS

Phenolic compounds were identified and quantified using UPLC-Q-TOF-MS (Waters, Milford, MA, USA) according to the protocol described by Żurek et al. [37]. The separation of individual phenols was performed at 50 °C, using a UPLC BEH C18 column (100 mm × 2.1 mm, 1.7 µm, Waters, Warsaw, Poland). The eluent was a mixture of water (Solvent A) and 40% acetonitrile in water, *v*/*v* (solvent B). The flow rate was kept constant at 0.35 mL/min. The solvent gradient was as follows: 0–8 min, 5–100% B, 8–9.5 min washing, and coming back to initial conditions. The injection volume was 5 μL. The following parameters were used for triple-quadrupole detection: gas flow con 100 L/h; voltage 30 V; capillary voltage 3.5 kV; source temperature 120 °C; desolvation temperature 350 °C; and desolvation gas flow 800 L/h. The quantification of polyphenolic compounds was performed by the use of internal standard method. Chlorogenic acid, quercetin 3-*O*-rutinoside, apigenin 8-*C*-glucoside, and kaempferol 3-*O*-glucoside were selected as internal standards of calibration for phenolic acids, quercetin, apigenin, and kaempferol derivatives, respectively. Standard curve calibrations were prepared in a concentration range 25–250 μg/mL of standard dissolved in 50% acetonitrile in water solution, with five different concentration levels (25, 50, 100, 150, 250 μg/mL). Triplicate injections were made for each level, and a weighted linear regression was generated. Concentrations of polyphenolics were calculated by preparing a calibration curve of mass concentration vs. peak area. For the linear regressions of the internal standards, *R*^2^ was 0.997, 0.999, 0.999, and 0.998 for chlorogenic acid, quercetin 3-*O*-rutinoside, apigenin 8-*C*-glucoside, and kaempferol 3-*O*-glucoside, respectively. Results are expressed in mg/100 g dw.

### 3.5. Determination of Antioxidant Activity

#### 3.5.1. ABTS^•+^ Radical Scavenging Activity

The scavenging activity of fruit and leaf extracts on ABTS^•+^ radicals was determined according to the method of Re et al. [38]. The plant extracts were mixed with ABTS^•+^ solution. After 6 min, the absorbance was measured at 734 nm. The results were expressed as Trolox Equivalent per g of dw (mmol TE/g dw).

#### 3.5.2. Determination of Copper Ion Reduction

The CUPRAC test was determined by method described by Apak et al. [39]. The plant extracts were mixed with 1.0 mL 10 mM copper chloride, 1.0 mL 7.5 mM neocuproine solution, and 2.0 mL 1 M acetate buffer. After 30 min, the absorbance was measured at 450 nm. The results were expressed as Trolox Equivalent (mmol TE/g dw).

#### 3.5.3. Chelating Ability of Ferrous Ion

The ability of the extracts to chelate iron ions (Fe^2+^) was assessed according to the method described by Mosmann [40]. The plant extracts were mixed with 0.4 mL of 0.1 mM iron II sulfate and 0.8 mL of 0.25 mM ferrozine solution. After 10 min, the absorbance was measured at 562 nm. The results were expressed as IC_50_.

#### 3.5.4. Superoxide Radical Scavenging Activity Assay

Superoxide radical scavenging activity was measured based on the method described by Robak and Gryglewski [41]. The plant extracts were mixed with 1.0 mL nitro blue tetrazolium, 1.0 mL β-nicotinamide adenine dinucleotide, and 1.0 mL phenazine methosulfate. After 5 min, the absorbance was measured at 560 nm. The results were expressed as IC_50_.

#### 3.5.5. Hydroxyl Radical Scavenging Activity Assay

Hydroxyl radical scavenging activity was evaluated by the method of Żurek et al. [33]. The plant extracts were mixed with 2.0 mL of 2-deoxyribose, 0.2 mL iron ammonium sulfate, 0.15 mL EDTA, 0.15 mL ascorbic acid, and 0.02 mL perhydrol. The solution was kept for 60 min at 37 °C, then 1.5 mL trichloroacetic acid and 1.0 mL thiobarbituric acid were added. After boiling and cooling to 37 °C, the absorbance was measured at 532 nm. The results were expressed as IC_50_.

### 3.6. Cell Culture

The human cancer cell lines breast adenocarcinoma (MCF-7), colon adenocarcinoma (Caco-2 and HT-29), and glioblastoma (U87MG) were obtained from the Sigma-Aldrich company (ECACC, Steinheim, Germany) and from the collection of the Nencki Institute of Experimental Biology, Polish Academy of Sciences, Warsaw, Poland. All cell lines were cultured in DMEM media supplemented with heat-inactivated fetal bovine serum and antibiotics. Cells were passaged with 0.25% trypsin-EDTA after washing with phosphate-buffered saline. All cultures were carried out at 37 °C and in a CO_2_ atmosphere (5%).

### 3.7. MTS Cell Viability Assay

Cell viability was assessed according to our previous reports, with some modifications [42]. The cell lines MCF-7, Caco-2, HT-29, and U87MG were seeded in 96-well microtiter plates at a density of 8.0 *×* 10^3^ cells/200 µL and incubated until they adhered at 37 °C and 5% CO_2_. The medium was then removed and the cells were treated with *E. japonica* leaf and fruit extracts at five concentrations (10, 100, 250, 500, and 750 µg/mL). After 48 h, the MTS test (Promega) was performed and the absorbance was measured at 490 nm (SmartReader 96, Edison, Accuris Instruments, Edison, NJ, USA). Untreated cells in medium were used as a control. The results are expressed as IC_50_.

### 3.8. Statistical Analysis

All analyses were performed in triplicate and are presented as mean ± SD. Duncan’s test (*p* < 0.05), Tukey’s HSD, Student’s t-test (*p* < 0.05; *p* < 0.01; *p* < 0.001), and Pearson’s correlation (*p* < 0.05; *p* < 0.01) were analyzed using Statistica 13.3 (StatSoft, Krakow, Poland).

## 4. Conclusions

Finally, it can be concluded that *E. japonica* is a promising source of phytochemicals with health benefits for future industrial research. Although fruits are more commonly used and appreciated as a food, the potential of leaves as a richer source of phenols could be considered. Thanks to the rich composition and antioxidant and chemopreventive properties, as well as great abundance in the Mediterranean region, *E. japonica* can not only promote human health but also improve bio-valorization and the environment.

## Figures and Tables

**Table 1:** The contents of total phenolics, flavonoids, and proanthocyanidins of *Eriobotrya japonica* fruit and leaf extracts.

	Total Phenolic Content (TPC)	Total Flavonoid Content (TFC)	Total Proanthocyanidin Content (TPA)
	(mg GAE/100 g dw)	(mg QE/100 g dw)	(mg CYE/100 g dw)
Fruits	6.05 ± 0.01	1.23 ± 0.00	1.19 ± 0.00
Leaves	47.99 ± 0.11	7.84 ± 0.00	6.34 ± 0.03

Abbreviations: CYE, equivalent of cyanidin chloride; dw, dry weight; GAE, equivalent of gallic acid; QE, equivalent of quercetin. Values are expressed as mean ± standard deviation (SD).

**Table 2:** Antioxidant activities of *Eriobotrya japonica* fruit and leaf extracts.

	ABTS^•+^ Radical Scavenging Activity	Determination of Copper Ion Reduction	Chelating Ability of Ferrous Ion	Superoxide Radical Scavenging Activity Assay	Hydroxyl Radical Scavenging Activity Assay
	(mmol TE/100 g dw)		IC_50_ (µg/mL)		
Fruits	17.38 ± 2.52	14.57 ± 0.09	>1000	>1000	>1000
Leaves	74.35 ± 3.78	62.01 ± 1.47	941.25 ± 9.07	244.30 ± 0.38	661.04 ± 0.97

Abbreviations: dw, dry weight; TE, Trolox Equivalent. Values are expressed as mean ± SD.

**Table 3:** Correlation between TPC, TFC, TPA, groups of polyphenolic compounds identified in the *Eriobotrya japonica* fruit extract and antioxidant (ABTS, CUPRAC), and antiproliferative (Caco-2, HT-29, MCF-7, U87MG) activity.

	ABTS	CUPRAC	Caco-2	HT-29	MCF-7	U87MG	TPC	TFC	TPA	Phenolic Acid	Flavonols	Flavones
ABTS	1.000	0.410	−0.379	0.568	0.532	0.148	−0.992 *	0.737	−0.362	−0.361	0.916	−0.500
CUPRAC		1.000	0.688	0.983 *	−0.553	−0.841	−0.777	0.918	−0.998 *	0.702	0.011	0.583
Caco-2			1.000	0.545	−0.985	−0.971	−0.078	0.345	−0.724	0.999 *	−0.717	0.990
HT-29				1.000	−0.394	−0.729	−0.878	0.974 *	−0.972	0.562	0.192	0.427
MCF-7					1.000	0.916	−0.093	−0.179	0.596	−0.981	0.825	−0.999 *
U87MG						1.000	0.313	−0.558	0.867	−0.975	0.530	−0.930
TPC							1.000	−0.962	0.743	−0.098	−0.638	0.056
TFC								1.000	−0.896	0.363	0.406	0.215
TPA									1.000	−0.730	0.039	−0.625
Phenolic acid										1.000	−0.703	0.987
Flavonols											1.000	−0.804
Flavones												1.000

(−) negative correlation, * significant differences at *p* < 0.05. TFC = Total Flavonoid Content; TPA = Total Proanthocyanidin Content; TPC = Total Phenol Content.

**Table 4:** Correlation between TPC, TFC, TPA, groups of polyphenolic compounds identified in the *Eriobotrya japonica* leaf extract and antioxidant (ABTS, CUPRAC), and antiproliferative (Caco-2, HT-29, MCF-7, U87MG) activity.

	ABTS	CUPRAC	ChA	O_2_˙^−^	OH^−^	Caco-2	HT-29	MCF-7	U87MG	TPC	TFC	TPA	Phenolic Acid	Flavonols	Flavones
ABTS	1.000	0.995 *	0.793	−0.704	0.711	0.782	−0.175	0.817	−0.508	−0.957	−0.137	0.056	0.997 *	0.629	0.511
CUPRAC		1.000	−0.640	−0.532	0.540	0.899	−0.386	0.671	−0.308	−0.871	−0.350	−0.162	0.995 *	0.783	0.687
ChA			1.000	0.991	−0.992	−0.240	−0.460	−0.999 *	0.927	0.935	−0.494	−0.653	0.063	−0.025	0.117
O_2_^•−^				1.000	−0.999 *	−0.109	−0.574	−0.984	0.969	0.879	−0.605	−0.748	0.195	0.107	0.248
OH^−^					1.000	0.118	0.567	0.986	−0.966	−0.883	0.598	0.742	−0.186	−0.098	−0.239
Caco-2						1.000	−0.750	0.280	0.138	−0.569	−0.724	−0.577	0.953	0.976	0.935
HT-29							1.000	0.423	−0.758	−0.116	0.999*	0.972	−0.914	−0.875	−0.995 *
MCF-7								1.000	−0.911	−0.948	0.457	0.621	−0.022	0.066	−0.076
U87MG									1.000	0.735	−0.785	−0.888	0.431	0.349	0.479
TPC										1.000	−0.154	−0.342	−0.294	−0.378	−0.241
TFC											1.000	0.981	−0.898	−0.856	−0.921
TPA												1.000	−0.796	−0.740	−0.828
Phenolic acids													1.000	0.996	0.998
Flavonols														1.000	0.989
Flavones															1.000

(−) negative correlation, * significant differences at *p* < 0.05. TFC = Total Flavonoid Content; TPA = Total Proanthocyanidin Content; TPC = Total Phenol Content.

**Table 5:** Individual phenolic compounds tentatively identified by UPLC-PDA-MS/MS in *Eriobotrya japonica* fruit and leaf preparations. Compound numbers correspond to those in Figure 2.

No.	Compound	Rt	λ_max_	[M − H]^−^ *m/z*		Content	
						Fruits	Leaves
		min	nm	MS	MS/MS	mg/100 g dw	
*Phenolic acids*							
**1**	Caffeic acid derivative	1.44	299 sh, 324	371	179	nd	0.740 ± 0.002 ^jk^
**2**	Caffeic acid derivative	1.79	299 sh, 324	371	179	nd	0.199 ±0.010 ^bc^
**3**	Caffeic acid derivative	2.11	299 sh, 324	371	179	nd	0.682 ± 0.014 ^ijk^
**4**	Neochlorogenic acid	2.23	299 sh, 324	353	191	nd	4.608 ± 0.110 ^r^
**5**	Caffeic acid derivative	2.40	299 sh, 327	297	179	nd	0.858 ± 0.010 ^l^
**6**	Caffeic acid derivative	2.58	299 sh, 327	297	179	nd	4.878 ± 0.03 ^s^
**7**	3-*O*-Coumaroylquinic acid	2.75	310	337	163, 119	nd	0.754 ± 0.007 ^k^
**8**	Chlorogenic acid	2.85	299 sh, 324	353	191	0.661 ± 0.001 ^f^	3.00 ± 0.062 ^p^
**9**	Cryptochlorogenic acid	2.98	299 sh, 324	353	191	nd	0.717 ± 0.013 ^ijk^
**10**	Caffeic acid *	3.19	299 sh, 324	179	135	0.275 ± 0.001 ^c^	0.653 ± 0.026 ^h^
**11**	Caffeic acid glucoside	3.30	299 sh, 324	341	179	nd	0.222 ± 0.042 ^cd^
**12**	Caftaric acid	3.47	299 sh, 324	311	179	nd	0.271 ± 0.14 ^de^
**13**	Ferulic acid	3.61	326	193	161	nd	0.827 ± 0.025 ^l^
**15**	Unidentified	4.39	295	217	-	nd	0.374 ± 0.032 ^f^
**17**	Caffeic acid derivative	4.70	299 sh, 324	481	179	0.089 ± 0.001 ^ab^	0.675 ± 0.005 ^ij^
**21**	Salvianolic acid B	5.09	282, 338	717	339	0.064 ± 0.01 ^ab^	nd
**25**	Rosmarinic acid *	5.64	329	359	161	2.031 ± 0.11 ^h^	1.152 ± 0.010 ^m^
**26**	Caffeic acid derivative	5.72	299 sh, 324	451	179	nd	0.153 ± 0.003 ^b^
**29**	Dicaffeoyl quinic acid	6.37	299 sh, 324	515	353, 179	nd	0.077 ± 0.004 ^a^
**31**	Caffeic acid derivative	6.71	299 sh, 324	373	179	0.033 ± 0.007 ^a^	nd
**32**	Caffeic acid derivative	6.81	299 sh, 324	373	179	0.096 ± 0.004 ^ab^	0.440 ± 0.033 ^g^
**33**	Feruloylquinic acid	7.10		367	193	nd	0.052 ± 0.004 ^a^
	Total					3.249 ± 0.001	21.335 ± 0.338
*Flavonols*							
**14**	Quercetin 3-*O*-xyloside *	3.90	255, 354	433	301	nd	0.191 ± 0.005 ^bc^
**16**	Kaempferol 3-*O*-sophoroside	4.47	260, 348	609	285	nd	0.299 ± 0.010 ^e^
**19**	Quercetin 3-*O*-malonylglucoside	4.93	255, 352	549	505, 301	0.131 ± 0.003 ^b^	1.838 ± 0.034 ^n^
**24**	Kaempferol 3-*O*-rutinoside *	5.55	264, 348	593	285	nd	0.287 ± 0.005 ^e^
	Total					0.131 ± 0.003	2.424 ± 0.189
*Flavones*							
**18**	Luteolin 7-*O*-glucoside *	4.82	253, 347	447	285	0.545 ± 0.045 ^e^	nd
**20**	Chrysoeriol rutinoside	5.00	253, 349	607	299	0.091 ± 0.003 ^ab^	2.846 ± 0.068 ^o^
**22**	Apigenin hexoside	5.48	267, 336	431	269	0.455 ± 0.011 ^d^	nd
**23**	Luteolin 7-*O*-malonylglucoside	5.53	253, 347	533	447	1.620 ± 0.067 ^g^	nd
**27**	Apigenin acetylhexoside	6.08	266, 336	473	269	0.222 ± 0.004 ^c^	nd
**28**	Apigenin acetylhexoside	6.21	266, 336	473	269	2.927 ± 0.161 ^i^	nd
**30**	Chrysoeriol	6.42	253, 349	299	284	0.046 ± 0.002 ^a^	nd
	Total					5.905 ± 0.279	3.037 ± 0.734

* Compounds identified by standards. Values are expressed as mean ± SD. Statistical significance (values marked with different letters, a–s) between identified associations was analyzed by Duncan’s test (*p* < 0.05). Abbreviations: [M − H]^−^, negative ion values; *m/z*, mass-to-charge ratio; Rt, retention time; UV–Vis, ultraviolet–visible; nd, not detected.

## Data Availability

Data are contained within the article and Supplementary Materials.

## Supplementary Materials

The following supporting information can be downloaded at: https://www.mdpi.com/article/10.3390/plants12183221/s1, Figure S1. Ethanol (30%, *v*/*v*) effect on the viability of breast adenocarcinoma (MCF-7), colorectal adenocarcinoma (Caco-2, HT-29), and glioblastoma (U87MG) cell lines. Table S1. Calibration curve parameters of the method developed for each standard.
