# Supplementary material for: Antioxidant and Antiproliferative Activities of Phenolic Extracts of Eriobotrya japonica (Thunb.) Lindl. Fruits and Leaves
Source: Plants (Basel). 2023 Sep 10;12(18):3221. doi: 10.3390/plants12183221 (PMC10536983; doi:10.3390/plants12183221)
Supplement: Supplementary file 1 [file plants-12-03221-s001.zip › plants-2561279-supplementary.pdf]

plants

MDPI

Supplementary material

Antioxidant and antiproliferative activities of phenolic extracts of *Eriobotrya japonica* (Thunb.) Lindl. fruits and leaves

Agata Maria Pawłowska [1], Natalia Żurek [1], Ireneusz Kapusta [1], Marinella De Leo [2,*], and Alessandra Braca[2]

[1] Department of Food Technology and Human Nutrition, College of Natural Sciences, University of Rzeszow, 4 Zelwerowicza St., 35-601 Rzeszow, Poland; nzurek@ur.edu.pl (N.Z.); ikapusta@ur.edu.pl (I.K.)
[2] Department of Pharmacy, University of Pisa, Via Bonanno Pisano 33, 56126 Pisa, Italy; marinella.deleo@unipi.it (M.D.L.); alessandra.braca@unipi.it (A.B.)
* Correspondence: marinella.deleo@unipi.it; Tel.: 0039-0502219706.

Content:

Supplementary Figure S1: Ethanol (30%, *v/v*) effect on the viability of breast adenocarcinoma (MCF-7), colorectal adenocarcinoma (Caco-2, HT-29), and glioblastoma (U87MG) cell lines.

Supplementary Table S1. Calibration curve parameters of the method developed for each standard.

S1 in black

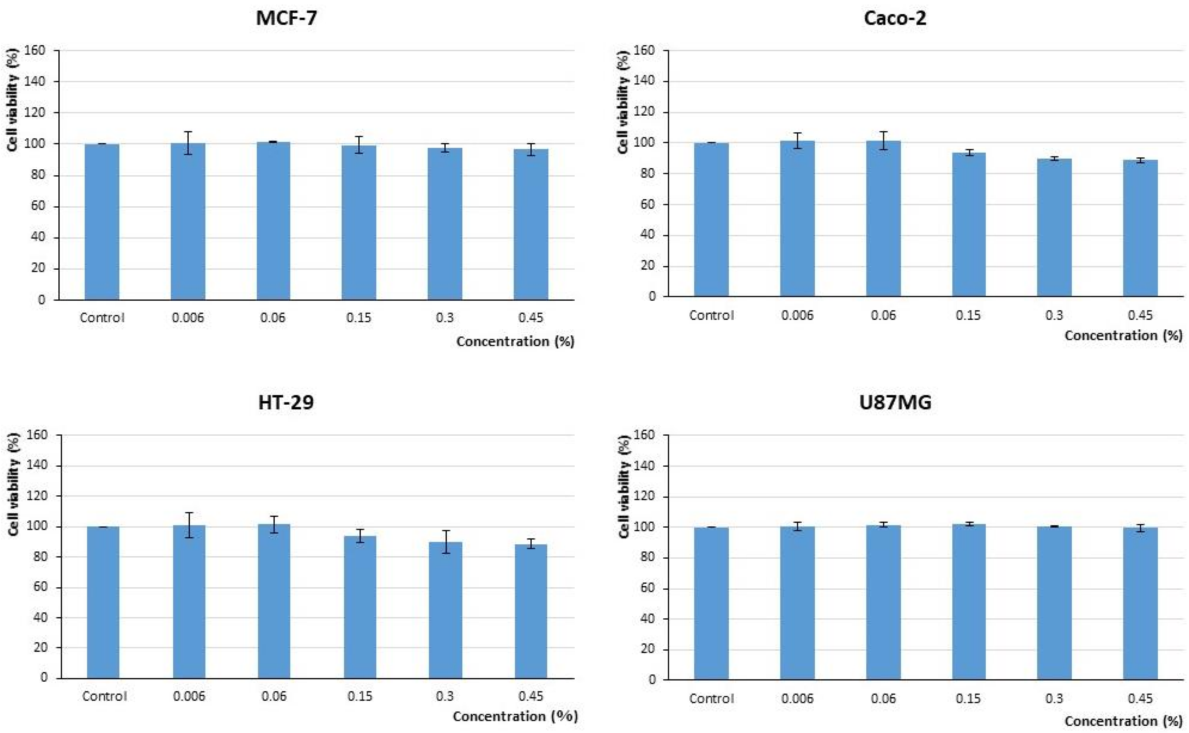

Figure S1. Ethanol (30%, *v*/*v*) effect on the viability of breast adenocarcinoma (MCF-7), colorectal adenocarcinoma (Caco-2, HT-29), and glioblastoma (U87MG) cell lines. Cells were treated for 48 h with different concentrations of ethanol (0.006-0.45%) deppending on the extracts concentration (see Figure 1). The viability was assayed by the MTS test. The number of viable control (non-treated) cells of each time point served as 100%. Graphs represent mean values ± SD from three independent experiments.

S1 in black

Table S1. Calibration curve parameters of the method developed for each standard.

Compound	Linearity range [µg/mL]	Regresion equation	LOD [µg/mL]	LOQ [µg/mL]
		Phenolic acids		
Chlorogenic acid	25-250	y = 6.16x10^{-5} – 3.40	0.055	0.168
		Flavonols		
Quercetin 3-*O*-rutinoside	25-250	y = 4.85x10^{-5} + 0.78	0.048	0.132
Kaempferol 3-*O*-glucoside	25-250	y = 1.09 x10^{-5} – 3.77	0.039	0.098
		Flavones		
Apigenin 8-*C*-glucoside	25-250	y = 1.33x10^{-5} – 4.02	0.012	0.036
